# Post-mortem lung biopsies in fatal Covid-19 acute respiratory distress syndrome: a prospective cohort study of 169 patients (HISTOCOVID)

**DOI:** 10.1186/s13613-025-01493-5

**Published:** 2025-06-11

**Authors:** Jean Morin, Christine Sagan, Morgane Pere, Riad Chelha, Pierre Yves Olivier, Georges Simon, Marion Rougon, Frédéric Pène, Alexis Ferre, Toufik Kamel, Alexandre Boyer, Julio Badie, Jan Hayon, Maxens Decavèle, Pierre Garçon, Jean-Christophe Richard, Laurent Argaud, Sami Hraiech, Johann Auchabie, Camille Foucault, François Legay, Paul-Marie Chanareille, Bertrand Souweine, Guillaume Géri, Agathe Delbove, Vincent Bonny, Pascal Beuret, Damien Vimpere, Gaëtan Plantefève, Emmanuel Canet

**Affiliations:** 1 Service de Médecine Intensive Réanimation, Centre Hospitalier Universitaire Hôtel-Dieu, Centre Hospitalier Universitaire de Nantes, 30 Bd. Jean Monnet, 44093 Nantes Cedex 1, France; 2 Service d’anatomie Pathologique, Centre Hospitalier Universitaire de Nantes, Nantes, France; 3 Direction de la Recherche, Plateforme de Méthodologie et Biostatistique, Centre Hospitalier Universitaire de Nantes, Nantes, France; 4 Service de Medecine Intensive et Réanimation, Hopital Privé Claude Galien, Quincy, France; 5 Service de Médecine Intensive Réanimation, CHU d’Angers, Angers, France; 6 Service de Réanimation Polyvalente, Centre Hospitalier de Troyes, Troyes, France; 7 Service de Médecine Intensive Réanimation, Centre Hospitalier Annecy Genevois, Annecy, France; 8 Service de Médecine Intensive and Réanimation, Hôpital Cochin, Assistance Publique-Hôpitaux de Paris, Institut Cochin, Université Paris Cité, Paris, France; 9 Service de Médecine Intensive Réanimation, CH de Versailles, Le Chesnay, France; 10 Service de Médecine Intensive Réanimation, CHR Orléans, Orléans, France; 11 Service de Médecine Intensive Réanimation, Université de Bordeaux, CHU de Bordeaux - Groupe Hospitalier Pellegrin, Bordeaux, France; 12 Service de Médecine Intensive Réanimation, Hôpital Nord Franche-Comté, Belfort, France; 13 Service de Médecine Intensive Réanimation, CHI Poissy-Saint-Germain-en-Laye, Poissy, France; 14 Service de Médecine Intensive Réanimation (Département R3S), APHP Sorbonne Université, Site Pitié-Salpêtrière, Paris, France; 15 Service de Médecine Intensive Réanimation, GHEF Site de Marne-La-Vallée, Jossigny, France; 16 Service de Médecine Intensive Réanimation, CHU Hôpital Lyon Sud (HCL), Lyon, France; 17 Service de Médecine Intensive Réanimation, CHU Edouard Herriot, Lyon, France; 18 Service de Médecine Intensive Réanimation, APHM CHU Nord, Marseille, France; 19 Service de Médecine Intensive Réanimation, CH Cholet, Cholet, France; 20 Service de Réanimation Polyvalente, CH Cahors - Hôpital Jean Rougier, Cahors, France; 21 Service de Médecine Intensive Réanimation, CH de Saint-Brieuc, Saint-Brieuc, France; 22 Service de Médecine Intensive Réanimation, GHPP Montélimar, Montélimar, France; 23 Service de Médecine Intensive Réanimation, CHU Clermont-Ferrand, Clermont-Ferrand, France; 24 Service de Médecine Intensive Réanimation, APHP, CHU Ambroise Pare, Boulogne-Billancourt, France; 25 Service de Réanimation Polyvalente, CHBA Vannes-Auray, Vannes, France; 26 Service de Médecine Intensive Réanimation, CHU Saint-Antoine, Paris, France; 27 Service de Réanimation Polyvalente, CH de Roanne, Roanne, France; 28 Service de Médecine Intensive Réanimation, Hôpital Necker, Assistance Publique-Hôpitaux de Paris, Université Paris Cité, Paris, France; 29 Service de Médecine Intensive Réanimation, Centre Hospitalier Victor Dupouy, Argenteuil, France

**Keywords:** Covid-19, Pathology, Lung biopsy, Acute respiratory distress syndrome, Diffuse alveolar damage

## Abstract

**Background:**

Refractory acute respiratory distress syndrome (ARDS) is the leading cause of death in patients with Covid-19. Large studies of lung pathology in patients who died of Covid-19-ARDS may help to understand the mechanisms of death and to guide further research.

**Methods:**

This prospective multicentre cohort study included 338 post-mortem, percutaneous, lung biopsies from 169 patients who died of Covid-19-ARDS between 22/04/2020 and 08/03/2021 in 26 intensive care units in France. The biopsies were done at the bedside by the intensivist immediately after death, using a 14G needle and following anatomical landmarks. A pathologist examined all biopsies, describing all elementary lesions and establishing a final histopathological diagnosis.

**Results:**

Lung parenchyma was evaluable in 155/169 (92%) patients. Early, proliferative-phase diffuse alveolar damage (DAD) was the most common finding (39%), followed by late proliferative-phase DAD (32%) and exudative-phase DAD (18%); fibrotic-phase DAD was present in three (2%) patients. Organising pneumonia (OP) and acute fibrinous and organising pneumonia (AFOP) were evidenced in 21 (13%) and 16 (9%) patients, respectively. Unclassified interstitial lesions were seen in 33 (21%) patients. Microthrombi were uncommon (6%).

**Conclusions:**

DAD was the most common pathological pattern, whereas collagen fibrosis and microthrombi were rare. A quarter of patients had evidence of OP or AFOP. This substantial prevalence of corticosteroid-sensitive patterns suggests that selected patients with refractory Covid-19-ARDS might benefit from higher doses or longer courses of corticosteroids.

Trial registration.

ClinicalTrials.gov NCT04675281. Registered 19 December 2020.

**Supplementary Information:**

The online version contains supplementary material available at 10.1186/s13613-025-01493-5.

## Introduction

Since December 2019, over 775 million cases of coronavirus disease-2019 (Covid-19) have been diagnosed worldwide, causing over 7 million deaths [1]. The SARS-CoV-2 virus responsible for Covid-19 can cause severe pneumonia with life-threatening acute respiratory distress syndrome (ARDS). Up to 15% of patients admitted for Covid-19 require intensive-care-unit (ICU) admission for Covid-19-ARDS, which carries a mortality rate of 30% to 60% [2–4].

ARDS is a clinical syndrome first described in 1967 [5] then defined by expert consensus in 1994, [6] with updates in 2012 [7] and 2023 [8]. Diffuse alveolar damage (DAD) is a pathological pattern combining hyaline membranes, interstitial oedema, and cell necrosis and proliferation, with or without fibrosis [9] DAD is considered the histological hallmark of ARDS. However, this view is based only on retrospective studies that included patients with ARDS due to various causes (including extra-pulmonary conditions) and were conducted over a long period during which invasive mechanical ventilation (iMV) practices and other components of ARDS management changed considerably [10, 11]. Studies of lung pathology in patients with Covid-19 have produced conflicting results, with some evidence that, in addition to DAD, pulmonary-vessel thrombosis and organising pneumonia (OP) or acute fibrinous and organising pneumonia (AFOP) may contribute to the development of refractory hypoxemia [12–16]. Most studies were done in a single centre and had small numbers of heterogeneous patients, raising concern about the interpretation and generalizability of their findings. Collecting data on lung histology in fatal Covid-19-ARDS is crucial to clarify the underlying pathophysiology and guide the assessment of new therapeutic interventions.

The objective of this large, prospective, multicentre study done in ICUs in France was to describe the lung pathology findings in a uniform population of patients who had died from Covid-19-ARDS. The lung samples were obtained using a standardized, post-mortem, percutaneous, needle-biopsy technique. We hypothesized that lung fibrosis would be uncommon.

## Methods

This report complies with STROBE guidelines [17]. The study is registered the 19 December 2020 on ClinicalTrials.gov under the number NCT04675281.

### Study design and patients

We conducted a prospective, multicentre, observational, cohort study in 26 ICUs in France (Supplementary e-appendix 1). All patients who died from Covid-19-ARDS in the ICUs of the participating centres between 22/04/2020 and 08/03/2021 were considered for enrolment. Inclusion criteria were age 18 years or older, signs and symptoms of lower-respiratory-tract infection (fever, dyspnoea, hypoxaemia requiring supplemental oxygen, and pulmonary infiltrates by chest radiography or computed tomography at ICU admission), positive polymerase chain reaction for SARS-Cov-2 on a naso-pharyngeal swab or pulmonary sample (endotracheal aspirate or broncho-alveolar lavage fluid), legal determination of death in compliance with French law during the same ICU admission, and next-of-kin informed of the study. Exclusion criteria were listing of the patient in the French opt-out registry for organ and tissue donation, pregnancy, age younger than 18 years, and adult under guardianship.

### Ethics and consent to participate

The study was approved by the French Biomedicine Agency (*Agence de la Biomédecine*) and French Ministry of Education and Research, on 24 April, 2020 (#2020–016). In compliance with French law, at the time death was declared, the French registry of persons unwilling to donate organs or tissues was examined to confirm that the deceased patient was not registered. In addition, family members were interviewed to check that the patient had not expressed an unwillingness to donate organs and/or tissues. During the same interview, the relatives were informed orally about the study and given a physical print-out of an information letter. The information delivered was documented in the medical chart by the local investigator. French law did not require informed consent from the families.

### Biopsy procedure, tissue processing, and histopathological evaluation

Percutaneous transthoracic lung biopsies were performed by the intensivist in charge of the patient immediately after death, using a 14G needle and following anatomical landmarks. Two biopsies per patient (one anterior and apical and one posterior and basal) were performed when possible, on the side most severely affected according to the most recent imaging studies (Supplementary e-appendix 2). All samples were fixed in 4% neutral formaldehyde for at least 48 h then embedded in paraffin wax at the pathology department of each participating centre. All biopsy samples were sent to the pathology department of the Nantes University Hospital, where they were evaluated by a single lung pathologist (CS) blinded to clinical data. For each biopsy, the pathologist described and interpreted all elementary lesions, using a predefined semi-quantitative histological scoring system, then determined the final histopathological diagnosis. When more than one biopsy was available for a given patient, the final histopathological diagnosis was based on findings from all biopsies. The pathologist used a standardized, web-based, electronic case-report form to record all elementary lesions and the final histopathological diagnosis for each patient.

### Data collection

For each patient, the data reported in the tables were extracted from the ICU records and entered by the local investigator at each centre into a standardized web-based electronic case-report form (Castor Electronic Data Capture System, CastorEDC, Amsterdam, The Netherlands). Gender was self-reported by the patients or relatives as male or female at ICU admission. ARDS was diagnosed according to the Berlin definition. [7] Refractory ARDS was defined as a fatal case of Covid-19 ARDS. The local investigators recorded whether possible or probable invasive pulmonary aspergillosis was diagnosed pre-mortem. Proven aspergillosis was defined as evidence of invasive aspergillosis by pathological examination of the post-mortem lung biopsies.

### Objectives

The primary objective of the study was to describe the histopathological features of post-mortem lung biopsies from patients who died in the ICU from Covid-19-ARDS. The secondary objectives were to describe differences in histopathological findings according to ICU length of stay, to report the incidence of potentially corticosteroid-sensitive lesions (OP or AFOP), and to assess the presence of irreversible collagen fibrosis.

### Statistical analysis

Descriptive statistics were computed for the baseline features in the overall population. Qualitative data were described as number (percentage) and compared across groups using the chi-square test or, if necessary, Fisher’s exact test. Quantitative data were described as mean ± standard deviation (SD) if normally distributed and as median [interquartile range] otherwise and were compared using Student’s *t* test or the Mann–Whitney test, respectively. Missing data were counted and reported. All analyses were performed using the available data, and no imputation methods were employed for missing values. The statistical analyses were done using SAS software version 9·4 (SAS Institute, Cary, NC). *P* values < 0.05 were taken to indicate significant differences.

### Role of the funding source

This study was funded by grants from two publicly funded non-profit sources, the *Agence Nationale de la Recherche* and the *Region Pays-de-Loire*, respectively. The sponsor was the Nantes University Hospital and had no role in designing the study; collecting, analysing, or interpreting the study data; writing the report; or deciding to submit the report for publication. All authors had full access to all the study data and accept final responsibility for the decision to submit for publication.

## Results

### Study population

We studied 169 patients who died from Covid-19-ARDS in the participating ICUs. Table 1 reports their main characteristics. Elderly males predominated and overweight, diabetes, and immunosuppression were the most common comorbidities. At ICU admission, all patients had clinical and radiological features of interstitial pneumonia. According to the Berlin definition, 149 (94·9%) patients developed severe and eight (5·1%) moderate ARDS; data on ARDS severity were unavailable for 12 patients. Table 2 reports the interventions used in the ICU. Most patients received extended, lung-protective, volume control ventilation. Median iMV duration was 17 [9–32] days. Veno-venous extra-corporeal membrane oxygenation was implemented in 14 patients, for 32·5 [14·0–39·0] days. Blood gas analysis was performed in 124 patients within 48 h before death and showed severe hypoxaemia (PaO_2_/FiO_2_, 72·8 [57·0–93·8]) and respiratory acidosis (pH, 7·3 [7·2–7·4]; and PCO_2_, 8·1 kPa [6·4–10·0]). First-line corticosteroid therapy was administered to 144 (90·0%) patients, for a median of 10 [9–11] days, and second-line corticosteroid therapy to 36 (25·0%) patients within 15 [11–20] days after first-line initiation. During the ICU stay, 106 (66·3%) patients developed at least one episode of ventilator-associated pneumonia, including nine (5·6%) diagnosed with possible or probable Covid-19-associated pulmonary aspergillosis. Pulmonary embolism or proximal venous thrombosis was diagnosed in 23 (14·7%) patients.

**Table 1:** Characteristics of the 169 patients

Patient characteristics	N (%) or Median [IQR]	n missing data
Age, years	72 [66–76]	8
Males	124 (73)	
Body mass index, kg/m^2^	29 [25–32]	14
History of smoking	65 (41)	12
Diabetes	53 (33)	8
History of respiratory disease^a^	37 (23)	8
Obstructive pulmonary disease^b^	20 (12)	
Obstructive sleep apnoea	19 (12)	
Other respiratory diseases^c^	5 (3)	
Charlson Comorbidity Index	4 [3–6]	8
Immunodeficiency	26 (16)	9
Corticosteroids or other immunosuppressants	10 (6)	
Haematological malignancy	9 (6)	
Solid organ transplantation	6 (4)	
Solid cancer	4 (3)	
Other	3 (2)	
Time from symptom onset to hospital admission, days	6 [4–9]	11
Time from hospital admission to ICU admission, days	1[0–4]	8
Time from hospital admission to intubation, days	4 [1–9]	18
Time from ICU admission to death, days	20 [12–32]	8
Primary cause of death^d^		8
Hypoxaemia and shock	57 (35)	
Hypoxaemia	54 (34)	
Shock	26 (16)	
Cardiac arrest	11 (7)	
Neurological failure	6 (4)	
Withdrawal of life-supporting interventions^e^	7 (4)	

*ICU* intensive care unit

^a^Some patients had more than one type of respiratory disease

^b^Chronic obstructive pulmonary disease, n = 14; Asthma, n = 5; Bronchiectasis, n = 1

^c^Restrictive pulmonary disease, n = 3; Interstitial lung disease, n = 1; Pulmonary hypertension, n = 1

^d^The primary cause of death was determined by the local investigator

^e^clinical frailty, n = 4; ventilator weaning failure, n = 1; advanced solid cancer, n = 1; not recorded, n = 1

**Table 2:** Management of the 169 patients in the intensive care unit

	N (%) or Median [IQR]	n missing data
Ventilation mode 48 h before death		14
Volume-control ventilation	127 (82)	
Airway pressure release ventilation	9 (6)	
Pressure support ventilation	5 (3)	
Pressure control ventilation	3 (2)	
Other modes^a^	11 (7)	
Ventilator settings for volume control ventilation (n = 127) 48 h before death		
FiO_2_, %	100 [80–100]	
Respiratory rate, cycles/minute	32 [30–35]	11
PEEP, cmH_2_O	8 [6–10]	10
Plateau pressure, cmH_2_O	31 [28–35]	35
Tidal volume, mL	390 [328–432]	11
Minute ventilation, L/min	12.5 [10·5–14·0]	24
Tidal volume/predicted body weight, mL/kg	5.9 [5·3–6·3]	12
Static compliance^b^, mL/cmH_2_O	18 [13–22]	35
Life-supporting interventions in addition to ventilation		
Prone positioning	135 (84)	8
Number of prone-position sessions/patient	4 [2–7]	1
Veno-venous ECMO	14 (9)	8
Neuromuscular blockers	143 (89)	8
Nitric oxide	14 (9)	8
Vasopressors	137 (86)	9
Renal replacement therapy	59 (58)	9

*PEEP* Positive end-expiratory pressure, *ECMO* Extracorporeal membrane oxygenation

^a^High-flow oxygen, n = 8; Non-invasive ventilation, n = 3

^b^computed as tidal volume/(plateau pressure—positive end-expiratory pressure)

### Lung biopsies

**Fig. 1:**
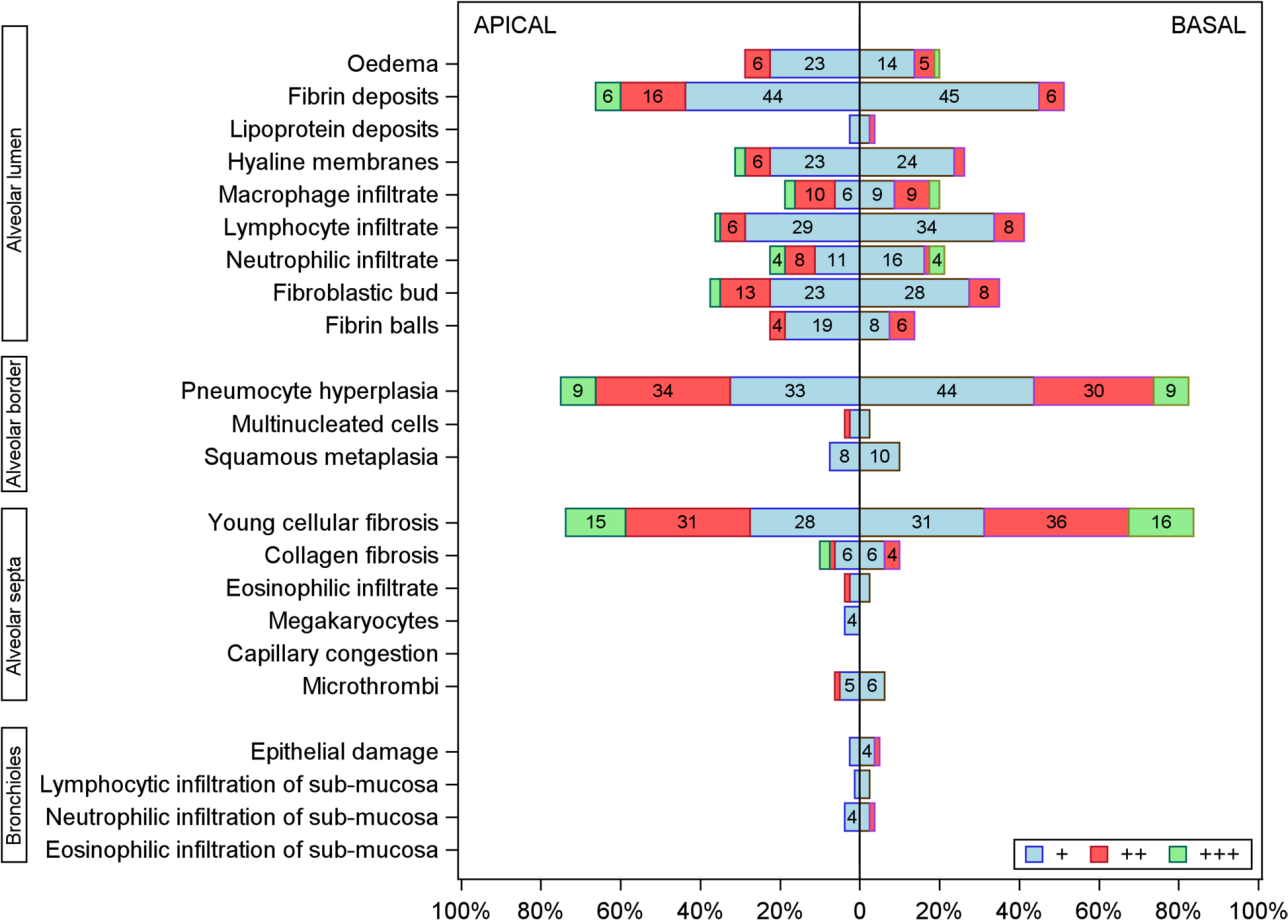
Histological findings in post-mortem apical and basal lung biopsies from patients who died from COVID-19-associated acute respiratory distress syndrome. Findings were categorised based on the percentage of tissue involved, as follows: absent (0%), focal (+), multifocal (+ +), or diffuse (+ + +)

The 169 patients had 338 lung biopsies (2·2 ± 1·0 per patient); 144 patients had both apical and basal biopsies and 25 had biopsies at either site. The lung parenchyma was evaluable in 155 (92%) patients. The distribution of elementary lesions is provided in e-Table 1 and e-Fig. 1. The most common elementary lesions were fibrin deposits (73·5%), hyaline membranes (37·7%), intra-alveolar fibroblastic buds (46·5%), and intra-alveolar fibrin balls (23·9%). Pneumocyte hyperplasia in alveolar borders was often seen (82·6%). Infiltration of alveolar septa by young cellular fibrosis (80·7%) and lymphocytes (33·7%) was also common, whereas collagen fibrosis (11·0%) and microthrombi (6·5%) were rare. The bronchioles were nearly normal. Figure 1 reports the numbers of patients with each elementary lesion. Although elementary lesions were similar in apical and basal biopsies, the numbers of each type correlated poorly between apical and basal sites in the 144 patients who had data from both sites (e-Table 2).

### Pathological diagnoses

Early proliferative-phase DAD was the most common pathological diagnosis (38·9%), followed by late proliferative-phase DAD (31·9%) and exudative DAD (18·5%). Only three (1·9%) patients had features of fibrotic-phase DAD. OP and AFOP were diagnosed in 21 (13·4%) and 16 (10·2%) patients, respectively. Unclassified interstitial pathology was recorded in 33 (21·0%) patients, and 15 (9·6%) patients had pathological findings of acute infectious bronchopneumonia. Other pathological diagnoses were found in 33 patients and were usually related to pre-existing lung disease (e-Table 3). Of these patients, two (1%) had proven invasive aspergillosis.

**Fig. 2:**
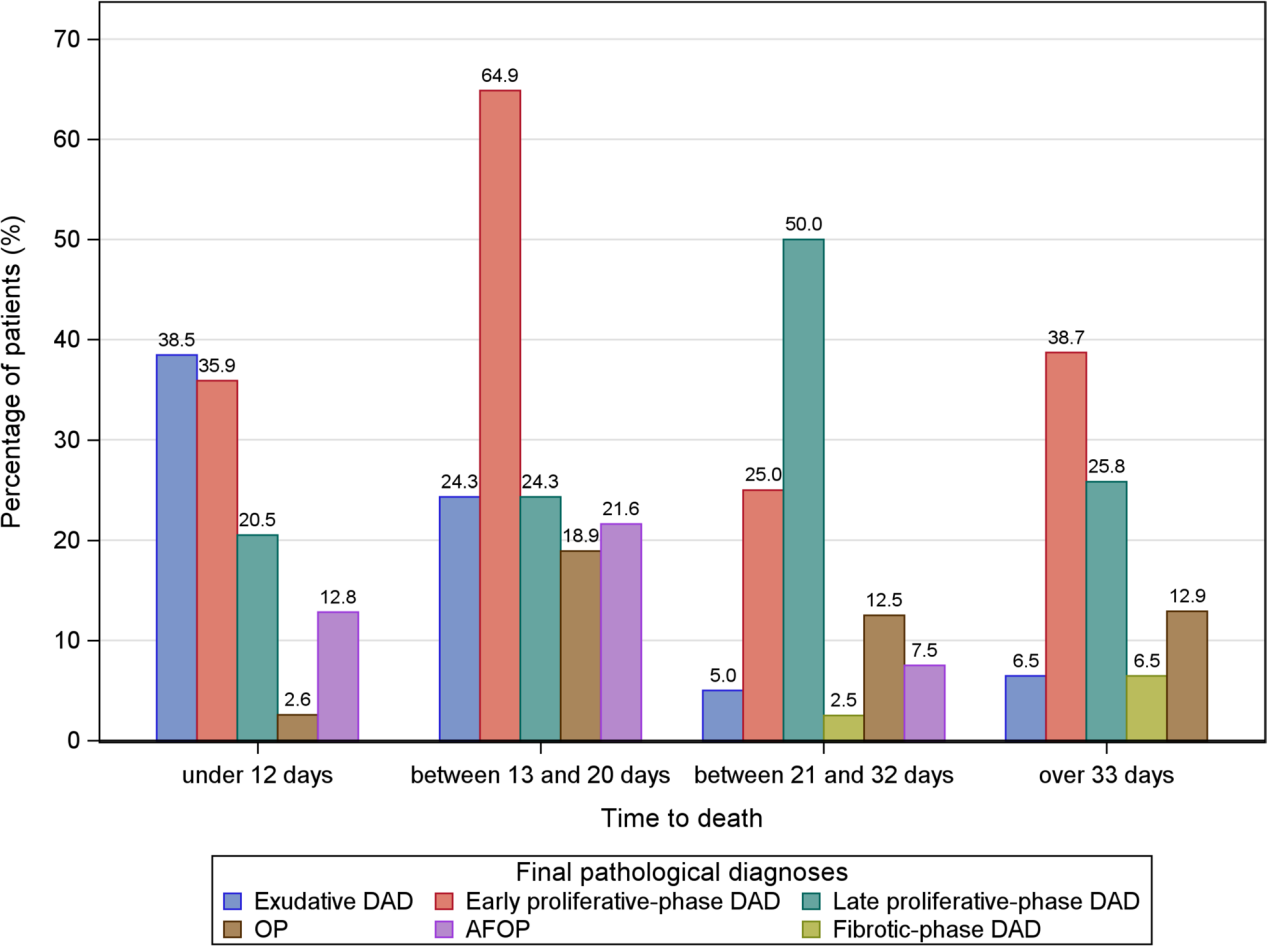
Distribution of final pathological diagnoses according to time from intensive-care-unit admission to death. *AFOP* Acute fibrinous and organizing pneumonia, *DAD* Diffuse alveolar damage, *OP* Organizing pneumonia

**Fig. 3:**
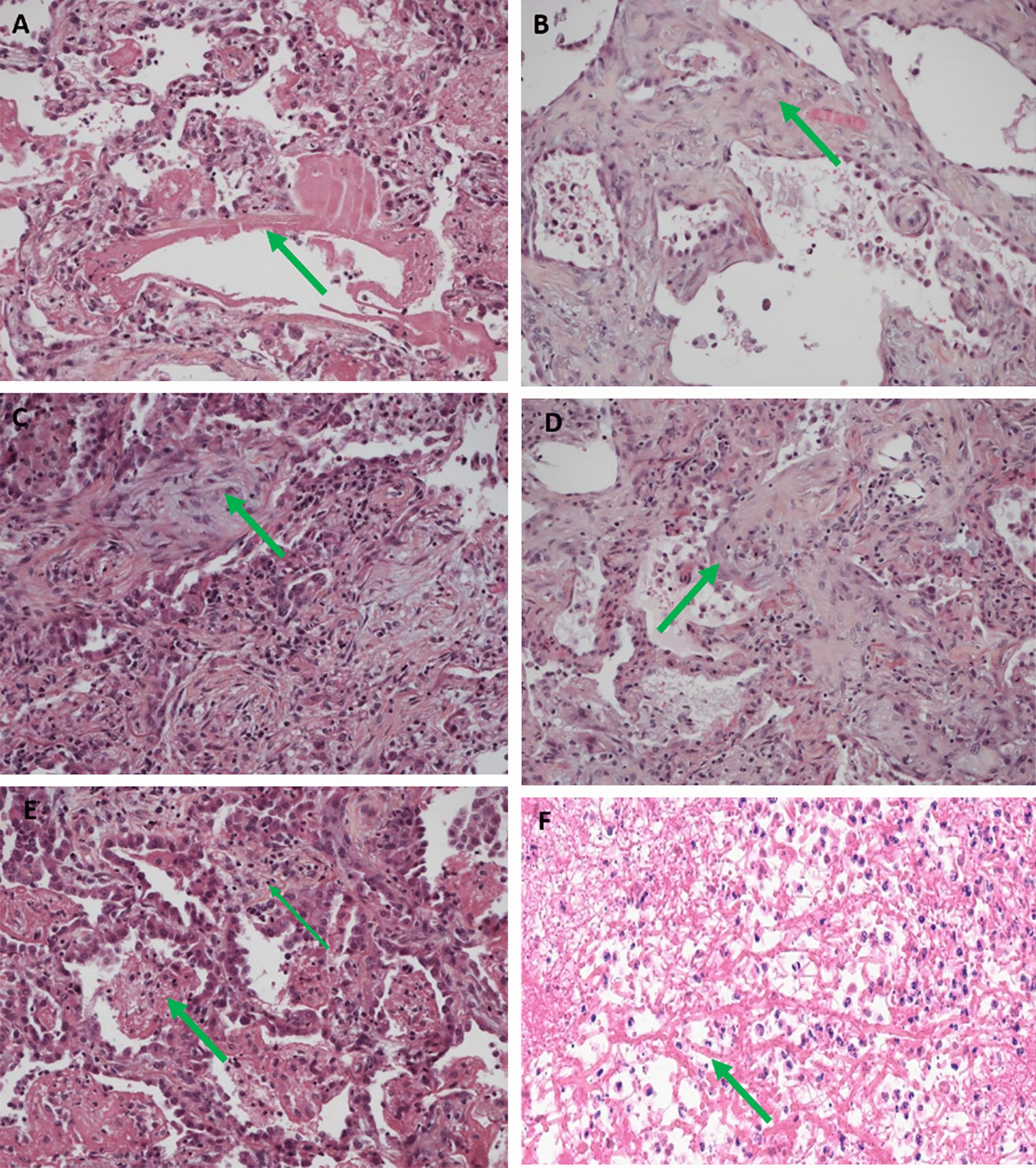
Examples of histological findings. Haematoxylin Eosin-Saffron stain. **A** Acute-phase diffuse alveolar damage (DAD): Hyaline membrane (arrow) (× 200). **B** Early proliferative-phase DAD: Organised hyaline membrane with septal thickening due to fibroblast proliferation (arrow) (× 200). **C** Late proliferative-phase DAD: The fibrosis is more severe and fibroblasts are proliferating within the alveoli (arrow) but the lung architecture is preserved (× 200). **D** Organizing pneumonia: Fibroblast proliferation in the alveolar spaces (arrow) (× 200). **E** Acute fibrinous and organizing pneumonia: Extensive intra-alveolar fibrin (fibrin balls, thin arrow) with early organisation (thick arrow) and pneumocyte hyperplasia (× 200). **F** Aggregates of *Aspergillus* hyphae (arrow) and neutrophils (× 400)

Figure 2 shows the distribution of the main pathological diagnoses according to time from ICU admission to death. Exudative DAD was mainly reported in patients who died within 12 days (*P* = 0·0003), early proliferative-phase DAD in those who died between 13 and 20 days (*P* = 0·0033), and late proliferative-phase DAD in those with longer disease durations (*P* = 0·016). OP was equally distributed across time-to-death groups (*P* = 0·13). AFOP occurred within the first three weeks (*P* = 0·019) (e-Table 4). Most patients had more than one pathological diagnosis (e-Fig. 2). Figure 3 shows examples of common histological findings.

## Discussion

### Key findings

In this large multicentre study, post-mortem percutaneous lung biopsies performed by the intensivist at the bedside using anatomical landmarks enabled parenchymal lung histopathological evaluation in over 90% of patients. Our examination of 338 lung biopsies from 169 patients who died in the ICU from COVID-19-ARDS demonstrated that early and late proliferative-phase DAD were the most common findings and predominated in those patients with long iMV durations. Importantly, although 90% of the patients received corticosteroid therapy, a quarter of patients had evidence of OP or AFOP. Finally, collagen fibrosis, microthrombi, and proven invasive pulmonary aspergillosis were uncommon.

### Comparison to previous studies

The primary cause of death in patients with Covid-19-ARDS is refractory hypoxaemia. However, data on lung pathology come only from anecdotal case-reports and small, usually single-centre, cohorts [16, 18, 19]. These studies consistently found a high prevalence of DAD. In our cohort, 70% of patients had DAD, with early and late proliferative-phase disease being the most common patterns. Several studies demonstrated microthrombi in 86% to 100% of patients, [18–21] compared to 24% described in patients with other causes of ARDS [10, 22]. In contrast, we evidenced microthrombi in only 6% of patients, consistent with the failure of therapeutic anticoagulation to improve Covid-19 outcomes [23]. Different SARS-CoV-2 variants, anticoagulation protocols, and steroid strategies across studies may have contributed to the wide variation in microthrombi prevalence. OP and AFOP have been reported previously in Covid-19-ARDS, [12] with no data on incidence. In our cohort, 23% of patients had histological features of OP or AFOP, usually in combination with DAD at various phases.

Pulmonary collagen fibrosis is considered the final result of non-resolving DAD in patients with prolonged lung injury and is generally viewed as irreversible and as making a large contribution to refractory hypoxaemia in fatal ARDS [24–26]. In a 2021 systematic review of lung pathology in Covid-19, organizing fibrosis and end-stage fibrosis were reported in 52% and 1% of cases, respectively, with great variability across studies [22]. Our finding of young cellular fibrosis in 81% of patients and of collagen fibrosis in only 11% suggests that irreversible pulmonary fibrosis may be uncommon in fatal Covid-19-ARDS.

Invasive pulmonary aspergillosis is a complication of severe Covid-19 and is associated with increased mortality [27]. Its incidence varies widely across studies, from 2·5% to 28%, reflecting the considerable difficulty in establishing the diagnosis and the infrequent use of diagnostic lung biopsy [27, 28]. Whereas nine patients in our cohort were given a clinical diagnosis of possible or probable invasive aspergillosis before death, only two patients had proven invasive aspergillosis by lung biopsy, highlighting the limitations of current diagnostic criteria. In another study, the pathological examination of lungs patients who died with Covid-19 and a clinical diagnosis of invasive aspergillosis obtained no evidence of the latter (29).

### Study implications

Our findings demonstrate that collecting large-scale histological data after death from Covid-19-ARDS is feasible and generates valuable information. Routine, bedside, post-mortem, lung biopsy should therefore be encouraged. A multicentre registry of lung histological data would help to elucidate the mechanisms of death from Covid-19-ARDS and to guide further research programmes.

We found that, in the era of protective lung ventilation, DAD remained the most common pathological lesion but that the chronic phase of irreversible, interstitial, collagen fibrosis was rare. Thus, in clinical practice, the irreversibility of lung injury cannot be definitively determined based on iMV parameters or imaging-study findings. Moreover, the meaningful prevalence of corticosteroid-sensitive patterns, namely, OP and AFOP, raises the hypothesis that selected patients with Covid-19-ARDS might benefit from higher doses or longer courses of corticosteroids. This hypothesis is supported by a trial showing a trend, although not a significant difference, towards better outcomes with higher dexamethasone doses (30). Additional research is required to investigate the effect of steroids on DAD and young cellular fibrosis. Further studies are also needed to investigate whether thoracic computed tomography can identify COVID-19 patients with corticosteroid-sensitive patterns.

Finally, the lower prevalence of proven invasive aspergillosis in our study than suspected clinically implies that current diagnostic algorithms for possible and probable aspergillosis may result in overdiagnosis.

### Strengths and limitations

Our study has a number of strengths. It is the largest multicentre prospective cohort of lung biopsies from patients with Covid-19-ARDS to date. The large sample size supports the accuracy of the prevalence estimates for the histological elementary lesions and diagnoses, and the large number of participating centres supports the generalisability of the findings. Second, to minimise bias, all biopsies were evaluated centrally by a single expert pulmonary pathologist, who was unaware of the clinical data and used a predefined scoring system. Third, we collected both apical and basal biopsies, thus obtaining information on lesion distribution throughout the lungs. Fourth, we compared four groups that differed regarding time to death. This analysis demonstrated significant differences across groups.

One limitation of our study is that the biopsies were obtained post-mortem. Our data may not apply to patients who survive Covid-19-ARDS. However, they provide important information on those patients with the most severe disease and can serve to generate hypotheses about the mechanisms of death from COVID-19-ARDS. Second, we used percutaneous needle biopsies guided by anatomical landmarks. Open surgical biopsies or post-mortem examination of the entire lungs would have provided a more comprehensive assessment. Nevertheless, 90% of the needle biopsies allowed evaluation of the lung parenchyma. Moreover, the transcutaneous needle-biopsy technique does not require specific training and allows the collection of large numbers of samples. Third, the evaluation of all samples by a single pathologist carries a theoretical risk of interpretation bias. However, the pathologist had extensive experience and expertise in lung pathology, used a predefined scoring system, and was blinded to clinical data.

## Conclusion

The predominant pattern of lung lesions in patients who died from Covid-19-ARDS was early and late proliferative-phase DAD. Fibroblastic tissue proliferation, OP, and AFOP were often evidenced, whereas microthrombi and collagen fibrosis were rare. The substantial proportion of patients with potentially corticosteroid-sensitive histological patterns supports further research aimed at identifying a sub-group of patients with refractory Covid-19-ARDS most likely to benefit from higher doses or longer durations of corticosteroid therapy.

## Supplementary Information

Supplementary material 1

## Data Availability

The datasets generated for this study will not be publicly available owing to the limitations of participant consent and approvals in place regarding data sharing between organisations involved in the study. The data will be held at the Nantes University Hospital, Nantes, France, and will be used internally for secondary purposes. Any requests for potential data sharing or collaboration should be made to the corresponding author and will be considered. The study tools, including the protocol, consent forms, definition of outcomes, and regulatory documents are available upon request.

## Abbreviations

AFOP: Acute fibrinous and organising pneumonia
ARDS: Acute respiratory distress syndrome
Covid-19: Coronavirus disease 2019
DAD: Diffuse alveolar damage
ICU: Intensive care unit
iMV: Invasive mechanical ventilation
OP: Organising pneumonia
SARS-CoV-2: Severe acute respiratory syndrome-coronavirus 2
