# Supplementary material for: Post-mortem lung biopsies in fatal Covid-19 acute respiratory distress syndrome: a prospective cohort study of 169 patients (HISTOCOVID)
Source: Ann Intensive Care. 2025 Jun 11;15:80. doi: 10.1186/s13613-025-01493-5 (PMC12158891; doi:10.1186/s13613-025-01493-5)
Supplement: Supplementary file 1 — Supplementary material 1 [file 13613_2025_1493_MOESM1_ESM.docx]

**SUPPLEMENTARY MATERIAL**

**Post-Mortem Lung Biopsies in Fatal Covid-19 Acute Respiratory Distress Syndrome: A Prospective Cohort Study of 169 Patients (HISTOCOVID)**

**Contents**

e-Appendix 1: List of the 26 participating intensive care units in France………………. p 1

e-Appendix 2: Supplementary Methods………………….……………………….. p 2

Lung biopsy procedure..…………………………….…………………………….. p 2

Data collection………………………………………….…………………………….. p 3

Definitions…………………………………………………………………………………p 4

Elementary histopathological lesions…………………………………………………… .p 4

Definitions of histopathological diagnoses………………………………………….…….p 4

e-Table 1: Elementary lung lesions seen in the 155 patients with evaluable biopsies……p 5

e-Figure 1: Elementary lung lesions found in the 155 patients with evaluable biopsies… p 5

e-Table 2: Concordance analysis between apical and basal elementary lung lesions…. p 7

e-Table 3: Histopathological diagnoses unrelated to Covid-19-ARDS………………. p 7

e-Table 4: Distribution of histopathological diagnoses according to time of death…. p 7

e-Figure 2: Distribution of the main histopathological diagnoses……………………… p 8

References………………………………………………………….……………… p 8

**e-Appendix 1: List of the 26 participating intensive care units in France**

| **Participating centres** | **Number of patients included**  **(total=169)** |
| --- | --- |
| Service de Médecine Intensive Réanimation, CHU de Nantes | 21 |
| Service de Médecine Intensive Réanimation, Centre Hospitalier Victor Dupouy, Argenteuil | 16 |
| Service de Médecine Intensive Réanimation, Hôpital Privé Claude Galien, Quincy-sous-Sénart | 15 |
| Service de Médecine Intensive Réanimation, CHU d’Angers | 14 |
| Service de Réanimation Polyvalente, Centre Hospitalier de Troyes | 12 |
| Service de Médecine Intensive Réanimation, Centre Hospitalier Annecy Genevois, Annecy | 10 |
| Service de Médecine Intensive Réanimation, CHU Cochin, Cochin | 8 |
| Service de Médecine Intensive Réanimation, CHR Orléans | 8 |
| Service de Médecine Intensive Réanimation, CH André Maginot, Versailles | 8 |
| Service de Médecine Intensive Réanimation, CHU Bordeaux | 7 |
| Service de Médecine Intensive Réanimation, Hôpital Nord Franche-Comté, Belfort | 6 |
| Service de Médecine Intensive Réanimation, CHU Hôpital Lyon Sud (HCL), Lyon | 5 |
| Service de Médecine Intensive Réanimation, GHEF site de Marne-La-Vallée, Jossigny | 5 |
| Service de Médecine Intensive Réanimation, CHU Pitié-Salpêtrière, Paris | 5 |
| Service de Médecine Intensive Réanimation, CHI Poissy-Saint-Germain-en-Laye | 5 |
| Service de Médecine Intensive Réanimation, CHU Edouard Herriot, Lyon | 4 |
| Service de Réanimation Polyvalente, CH Cahors - Hôpital Jean Rougier, Cahors | 3 |
| Service de Médecine Intensive Réanimation, CH Cholet, Cholet | 3 |
| Service de Médecine Intensive Réanimation, APHM CHU Nord, Marseille | 3 |
| Service de Médecine Intensive Réanimation, CHU Ambroise Paré, Boulogne | 2 |
| Service de Médecine Intensive Réanimation, CHU Clermont-Ferrand | 2 |
| Service de Médecine Intensive Réanimation, GHPP Montélimar | 2 |
| Service de Médecine Intensive Réanimation, CH de Saint-Brieuc | 2 |
| Service de Médecine Intensive Réanimation, CH de Roanne | 1 |
| Service de Médecine Intensive Réanimation, CHU Saint-Antoine, Paris | 1 |
| Service de Réanimation Polyvalente, CHBA Vannes-Auray, Vannes | 1 |

**e-Appendix 2: Supplementary Methods**

***Lung-biopsy procedure***

Transcutaneous lung biopsies were performed by the intensivist in charge of the patient as soon as possible after death. The protocol below was provided to all participating centres.

*Preparation:*

- Patient in the supine position
- Maintain mechanical ventilation (positive end-expiratory pressure set at 10 cmH_2_O, maintain tidal volume); biopsy after extubation is also possible
- Select the most severely affected lung (right or left) based on intensivist expertise and most recent imaging study (most severely affected area)
- Gowning and gloving according to local hygiene rules
- Use of the transthoracic biopsy needle available on site (e.g., semi-automatic 14G Tru-Cut^®^ (Temno, Milan, Italy) or equivalent)

Two biopsies per patient were to be performed when possible.

- *Anterior apical biopsy*

Second intercostal space (ICS), needle perpendicular to the skin, 5 cm lateral to the sternum and midway between the sternal notch and the nipple. Multiple passes in a wheel-spoke pattern according to the visual quality of the sample to ensure the recovery of at least one high-quality sample.


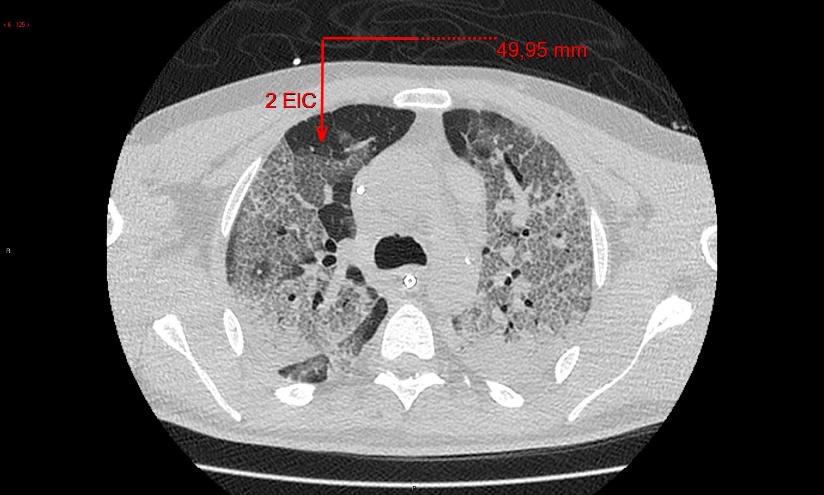

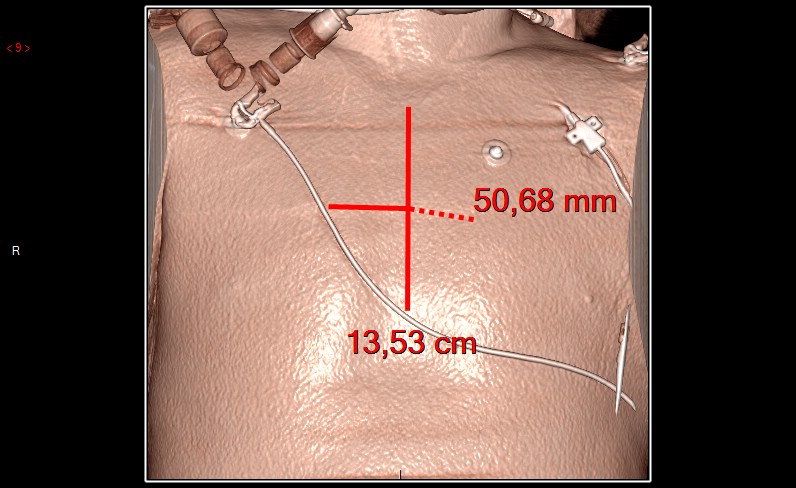


- *Posterior basal biopsy*

**Fifth ICS, needle directed towards the thoracic spine (posterior shot), at the level of the nipple on the axillary line.** Multiple passes in a wheel-spoke pattern according to the visual quality of the sample to ensure the recovery of at least one high-quality sample.


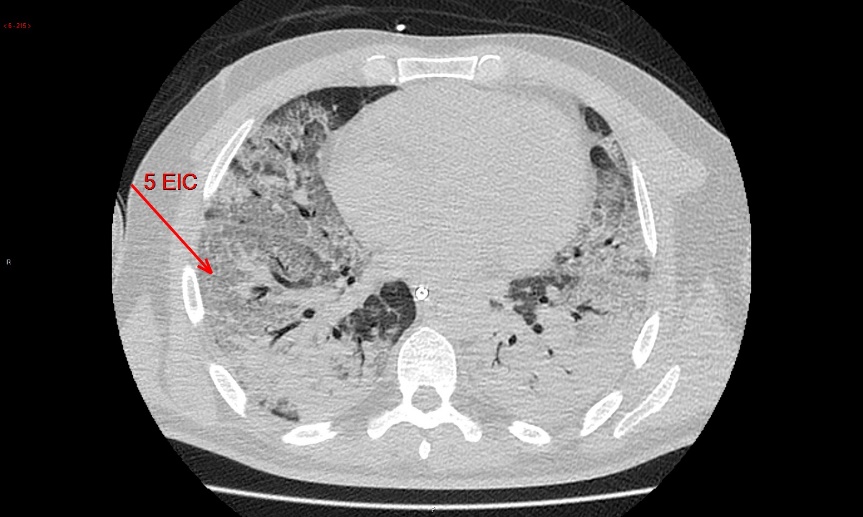

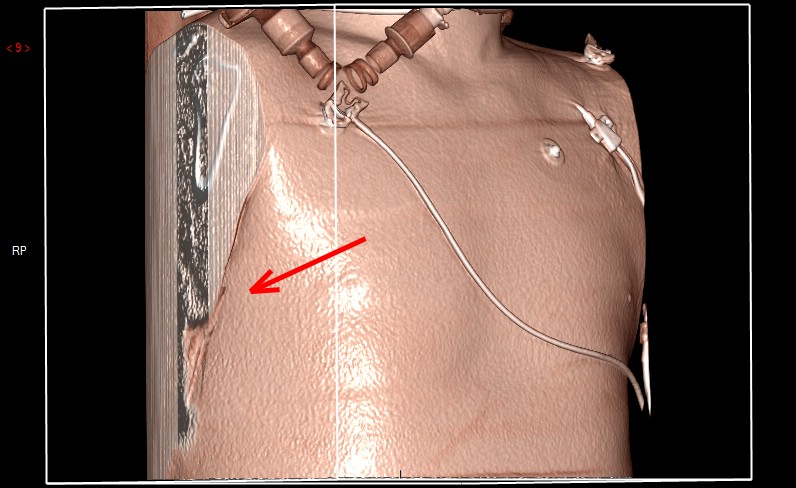


All samples were fixed in 4% neutral formaldehyde for at least 48 hours then embedded in paraffin wax at the pathology department of each participating centre. All biopsies were then sent to the pathology department of the Nantes University Hospital for evaluation by a single experienced pathologist.

***Data collection***

For each patient who died from Covid-19-ARDS, the data were collected retrospectively from the medical charts and entered by the local investigator into an electronic case-report form (Castor Electronic Data Capture System, CastorEDC, Amsterdam, The Netherlands). The collected data were age, sex as reported by the patient or family, body mass index, comorbidities and Charlson Comorbidity Index,^1^ underlying immunodeficiency (HIV infection, solid organ transplantation, haematological malignancy or solid cancer (new diagnosis or current progression or in remission for less than five years), chronic treatment with corticosteroids or other immunosuppressive drugs, or known primary immunodeficiency), SARS-CoV-2 infection characteristics, bacterial co-infection if present, date of ICU admission, baseline characteristics at ICU admission, treatments in the ICU (first-line and second-line corticosteroid therapy, antiviral drugs, immunomodulators, and vasopressors and/or inotropes), and Berlin criteria for ARDS met during the ICU stay^2^ with the duration and severity. Acute kidney injury was defined according to KDIGO criteria^3^ and the need for renal replacement therapy was recorded. The use of inhaled nitric oxide, prone positioning, and extracorporeal membrane oxygenation during the ICU stay was also collected. The following pulmonary complications during the ICU stay were collected: pulmonary embolism, pneumothorax or pneumomediastinum, pleural effusion, and nosocomial infections (ventilator-associated pneumonia [VAP] and invasive fungal infection, with microbiological documentation). The ventilator settings (mode, tidal volume, respiratory rate, peak inspiratory pressure, plateau pressure, positive end-expiratory pressure, and fraction of inspired oxygen) and arterial blood gas results during the 48 hours preceding death were collected. Findings from the last chest radiograph or computed tomography scan before death were recorded when available. The ICU and hospital stay lengths were also recorded. The cause of death was classified as follows by the local investigator: hypoxaemia, shock, hypoxaemia and shock, cardiac arrest, neurological failure, or other.

***Definitions***

OP and AFOP were classified as potentially corticosteroid-sensitive patterns based on the existing literature and the current treatment recommendations (1-5).”

1/ Lazor R, Cryptogenic organizing pneumonia. Characteristics of relapses in a series of 48 patients. The Groupe d'Etudes et de Recherche sur les Maladies "Orphelines" Pulmonaires (GERM"O"P), Am J Respir Crit Care Med. 2000 Aug;162(2 Pt 1):571-7. doi:10.1164/ajrccm.162.2.9909015.

2/ Cordier J.F. Cryptogenic organising pneumonia. Eur Respir J. 2006;28(2):422–446.

3/ Bradley B., Branley H.M., Egan J.J., et al. Interstitial lung disease guideline: the British Thoracic Society in collaboration with the Thoracic Society of Australia and New Zealand and the Irish Thoracic Society. Thorax. 2008;63(suppl 5):v1–v58.

4/ Copin M-C, Parmentier E, Daburcq T, Poissy J, Mathieu D; The Lille COVID-19 and Anatomopathology Group. Time to consider histologic pattern of lung injury to treat critically ill patients with COVID-19 infection. Intensive Care Med. 2020;46:1124–1126. doi: 10.1007/s00134-020-06057-8

5/ Cherian S, Algorithmic Approach to the Diagnosis of Organizing Pneumonia: A Correlation of Clinical, Radiologic, and Pathologic Features. Chest. 2022 Jul;162(1):156-178. doi: 10.1016/j.chest.2021.12.659. Epub 2022 Jan 14

VAP was defined as hospital-acquired pneumonia diagnosed after at least 48 h of invasive mechanical ventilation or within 48 hours after extubation. The diagnosis of VAP was established by the intensivist in charge of the patient using criteria recommended by the European Centre for Disease Prevention and Control: new radiological lung infiltrates combined with at least one systemic sign (temperature >38.3°C not due to another cause and/or leucocyte count <4000/mm^3^ or >12 000/mm^3^) and with one or more respiratory signs (new onset of purulent sputum or change in character of sputum and/or worsening gas exchange plus at least one positive microbiological sample [quantitative cultures of a distal blind protected-specimen brush, with a threshold of 10^3^ colony-forming units/mL; or of a bronchoalveolar-lavage specimen, with a threshold of 10^4^ colony-forming units/mL; or of an endotracheal aspirate, with a threshold of 10^6^ colony-forming units/mL; or positive culture of a pleural-fluid specimen).^4^

Finally, we recorded whether the local investigator made a pre-mortem diagnosis of possible or probable pulmonary invasive aspergillosis.

***Elementary histopathological lesions***

A single pathologist with extensive experience and expertise in the field of lung pathology evaluated all biopsies. Paraffin-embedded sections 3 μm in thickness were stained with haematoxylin eosin-saffron.

All biopsies were assessed using a predefined semi-quantitative scoring system. Findings were categorised based on the percentage of tissue involved, as follows: absent (0%), focal (+), multifocal (++), or diffuse (+++). The lesions types are listed in e-Table 1 and e-Figure 1. For patients with multiple biopsies, the most prominent finding available is reported. The data are from the 155/169 patients who had at least one biopsy allowing evaluation of the lung parenchyma.

***Definitions of histopathological diagnoses***

When more than one biopsy per patient was available, the final histopathological diagnosis was based on all available lung biopsies.

- ***Diffuse alveolar damage (DAD)***

Criteria for DAD were hyaline membranes plus at least one of the following: oedema, cell necrosis or proliferation, or fibrosis. Acute exudative-phase DAD was defined as predominance of pneumocyte necrosis, intra-alveolar hyaline membranes, capillary congestion, and intra-alveolar oedema. Early proliferative-phase DAD was defined as proliferation of alveolar type 2 cells, interstitial proliferation of fibroblasts and myofibroblasts, or young organising interstitial cellular fibrosis. Late proliferative-phase DAD was defined as dense collagen deposition in airspace granulation tissue, alveolar duct (ring) fibrosis, and an interstitial-pneumonia pattern (interstitial-appearing granulation tissue secondary to parenchymal collapse). Chronic fibrotic-phase DAD was defined as the presence of collagen fibrosis.^5–7^

- ***Acute fibrinous and organizing pneumonia (AFOP), and organizing***

***pneumonia (OP)***

AFOP was defined as a predominance of fibrin balls (organising intra-alveolar fibrin) within the alveolar spaces, with organisation resulting from fibroblast migration and secretion of young collagen within fibrin aggregates.^8,9^

OP was defined as intraluminal tufts of plump fibroblasts and young/immature collagen tissue within alveolar ducts and distal airspaces.^7^

- ***Acute infectious bronchopneumonia***

Acute pneumonia was diagnosed when intense neutrophilic infiltration of the interstitium and/or intra-alveolar spaces was found.

- ***Proven invasive pulmonary aspergillosis***

Invasive pulmonary aspergillosis was considered proven when tissue microscopy showed invasive growth of septate fungal hyphae.^10^

**e-Table 1: Elementary lung lesions seen in the 155 patients with evaluable biopsies**

| **Lesion type** | **Absent**  **n (%)** | **Focal (+)**  **n (%)** | **Multifocal (++)**  **n (%)** | **Diffuse (+++)**  **n (%)** |
| --- | --- | --- | --- | --- |
| **Alveolar lumen** |  |  |  |  |
| Oedema | 136 (87.8) | 11 (7.1) | 6 (3.9) | 2 (1.3) |
| Fibrin deposits | 41 (26.5) | 89 (57.4) | 20 (12.9) | 5 (3.2) |
| Lipoprotein deposits | 148 (96.1) | 5 (3.3) | 1 (0.7) | 0 (0) |
| Hyaline membranes | 96 (62.3) | 42 (27.3) | 13 (8.4) | 3 (2) |
| Macrophage infiltrate | 125 (80.7) | 12 (7.7) | 13 (8.4) | 5 (3.2) |
| Lymphocytic infiltrate | 139 (89.7) | 12 (7.7) | 4 (2.6) | 0 (0) |
| Neutrophilic infiltrate | 125 (80.7) | 16 (10.3) | 7 (4.5) | 7 (4.5) |
| Eosinophilic infiltrate | 154 (99.4) | 1 (0.7) | 0 (0) | 0 (0) |
| Fibroblastic bud | 83 (53.6) | 44 (28.4) | 25 (16.1) | 3 (1.9) |
| Fibrin balls | 118 (76.1) | 27 (17.4) | 10 (6.5) | 0 (0) |
| **Alveolar border** |  |  |  |  |
| Type 2 pneumocyte hyperplasia | 27 (17.4) | 49 (31.6) | 64 (41.3) | 15 (9.7) |
| Presence of multinucleated cells | 149 (96.1) | 5 (3.2) | 1 (0.7) | 0 (0) |
| Squamous metaplasia | 134 (86.5) | 16 (10.3) | 5 (3.2) | 0 (0) |
| **Alveolar septa** |  |  |  |  |
| Oedema | 122 (78.7) | 26 (16.8) | 7 (4.5) | 0 (0) |
| Young cellular fibrosis | 30 (19.4) | 35 (22.6) | 56 (36.1) | 34 (21.9) |
| Collagen fibrosis | 137 (89) | 8 (5.2) | 4 (2.6) | 5 (3.3) |
| Neutrophilic infiltrate | 138 (89.6) | 14 (9.1) | 1 (0.7) | 1 (0.7) |
| Eosinophilic infiltrate | 149 (96.8) | 4 (2.6) | 1 (0.7) | 0 (0) |
| Lymphocytic infiltrate | 99 (64.3) | 41 (26.6) | 13 (8.4) | 1 (0.7) |
| Presence of megakaryocytes | 151 (98.1) | 3 (2) | 0 (0) | 0 (0) |
| Capillary congestion | 154 (100) | 0 (0) | 0 (0) | 0 (0) |
| Microthrombi | 144 (93.5) | 9 (5.8) | 1 (0.7) | 0 (0) |
| **Bronchioles** |  |  |  |  |
| Epithelial damage | 146 (94.8) | 6 (3.9) | 2 (1.3) | 0 (0) |
| Lymphocytic infiltration of the submucosa | 151 (98.1) | 3 (2) | 0 (0) | 0 (0) |
| Neutrophilic infiltration of the submucosa | 150 (97.4) | 3 (2) | 1 (0.7) | 0 (0) |
| Eosinophilic infiltration of the submucosa | 154 (100) | 0 (0) | 0 (0) | 0 (0) |

**e-Figure 1: Elementary lung lesions found in the lungs of the 155 patients with evaluable biopsies**


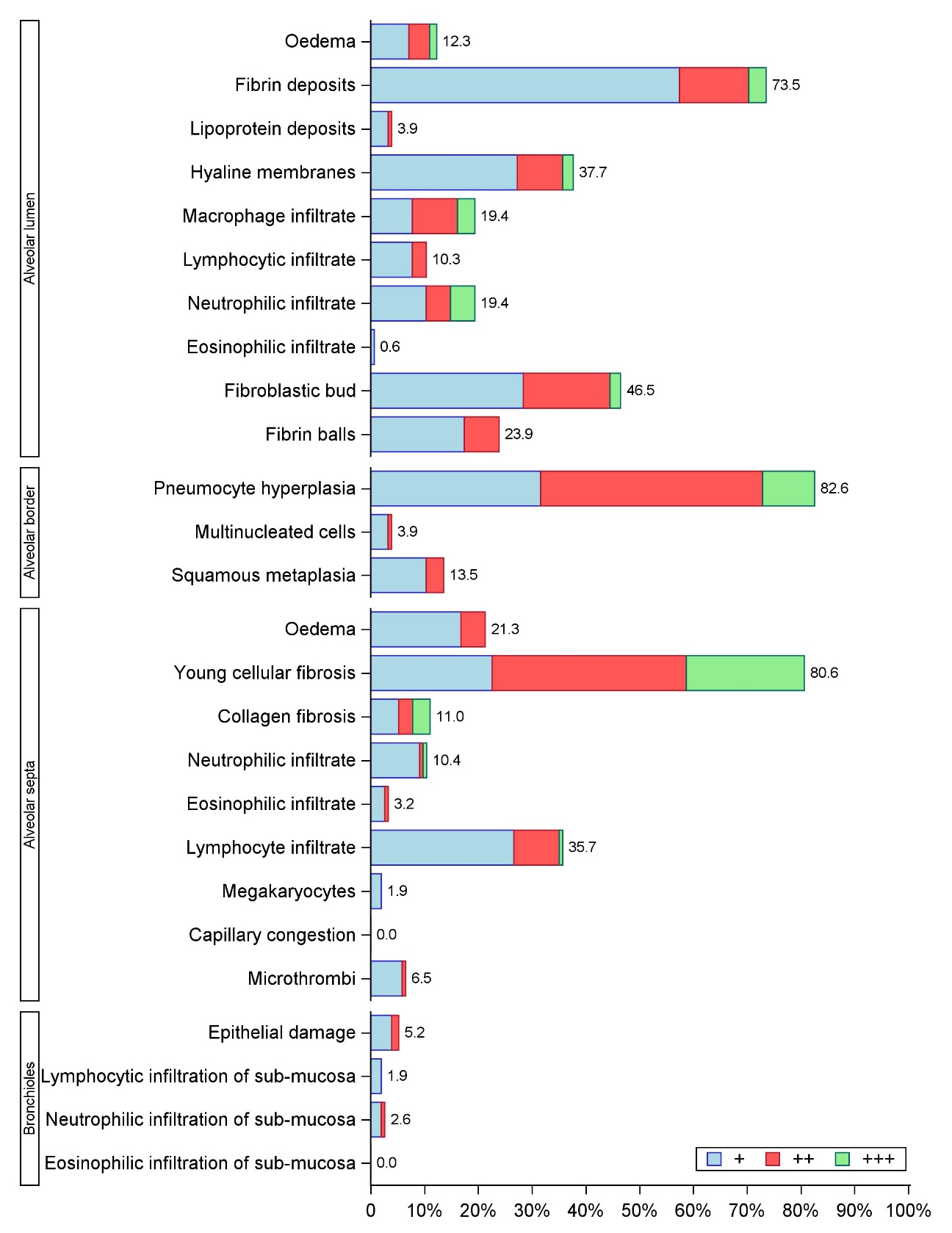


**e-Table 2: Concordance analysis between apical and basal elementary lung lesions in the 144 patients with evaluable biopsies from both locations**

| **Lesion types** | **Kappa coefficient** | **95% confidence interval** | |
| --- | --- | --- | --- |
| **Alveolar lumen** |  |  |  |
| Oedema | 0.16 | -0.09 | 0.42 |
| Fibrin deposition | 0.01 | -0.16 | 0.18 |
| Lipoprotein deposits | -0.03 | -0.06 | 0.00 |
| Hyaline membranes | 0.17 | -0.01 | 0.35 |
| Macrophagic infiltrate | 0.48 | 0.27 | 0.69 |
| Lymphocytic infiltrate | 0.34 | 0.03 | 0.65 |
| Neutrophilic infiltrate | 0.33 | 0.10 | 0.56 |
| Fibroblastic bud | 0.38 | 0.20 | 0.55 |
| Fibrin balls | 0.21 | -0.02 | 0.43 |
| **Alveolar border** |  |  |  |
| Type 2 pneumocyte hyperplasia | 0.24 | 0.08 | 0.40 |
| Presence of multinucleated cells | -0.03 | -0.06 | 0.00 |
| Squamous metaplasia | 0.37 | 0.03 | 0.72 |
| **Alveolar septa** |  |  |  |
| Oedema | 0.27 | 0.01 | 0.52 |
| Young cellular fibrosis | 0.18 | 0.03 | 0.33 |
| Collagen fibrosis | 0.39 | 0.14 | 0.64 |
| Neutrophilic infiltrate | 0.47 | 0.11 | 0.82 |
| Eosinophilic infiltrate | -0.02 | -0.05 | 0.01 |
| Lymphocytic infiltrate | 0.34 | 0.14 | 0.54 |
| Microthrombi | 0.36 | -0.04 | 0.76 |
| **Bronchioles** |  |  |  |
| Epithelial damage | 0.31 | -0.18 | 0.80 |
| Lymphocytic infiltrate in the sub-mucosa | -0.02 | -0.04 | 0.01 |
| Neutrophilic infiltrate in the sub-mucosa | 0.65 | 0.21 | 1.00 |

**e-Table 3: Histopathological diagnoses unrelated to Covid-19-ARDS, established in 33 patients**

| **Diagnosis** | **N (%)** |
| --- | --- |
| Minimal or no lung injury | 8 (24) |
| Tobacco-related disease | 4 (12) |
| Bronchiolar injury | 3 (9) |
| Ischaemic necrosis with *Aspergillus* | 2 (6) |
| Pre-existing interstitial lung disease | 2 (6) |
| Hepatocarcinoma | 1 (3) |
| Siderophage alveolitis | 1 (3) |
| Pleural fibrosis | 1 (3) |
| Herpes simplex virus infection | 1 (3) |
| Haematological malignancy | 1 (3) |
| Fibrinous pneumonia | 1 (3) |
| Lung sample too small to establish a final histopathological diagnosis | 8 (24) |

**e-Table 4: Distribution of histopathological diagnoses according to time of death in 161 patients; the data are n (%)**

|  | **≤12 days N=41** | | **13–20 days N=40** | **21–32 days N=40** | **>32 days N=40** | ***p* value** |
| --- | --- | --- | --- | --- | --- | --- |
| Acute exsudative DAD | 15 (38.5) | | 9 (24.3) | 2 (5.0) | 2 (6.1) | 0.0003 |
| Early proliferative-phase DAD | 14 (35.9) | | 24 (64.9) | 10 (25.0) | 12 (36.4) | 0.0033 |
| Late proliferative-phase DAD | 8 (20.5) | | 9 (24.3) | 20 (50.0) | 8 (24.2) | 0.016 |
| Fibrotic-phase DAD | 0 (0) | | 0 (0) | 1 (2.5) | 2 (6.1) | 0.17 |
| Organising pneumonia | 1 (2.6) | | 7 (18.9) | 5 (12.5) | 4 (12.1) | 0.13 |
| AFOP | 5 (12.8) | | 8 (21.6) | 3 (7.5) | 0 (0) | 0.019 |
| Unclassified interstitial pathology | 0 (0) | | 12 (32.4) | 12 (30.0) | 5 (15.2) | 0.0008 |
| Acute infectious bronchopneumonia | 2 (5.1) | | 2 (5.4) | 5 (12.5) | 4 (12.1) | 0.54 |
| Other diagnoses | 11 (28.2) | | 5 (13.5) | 8 (20.0) | 9 (27.3) | 0.39 |
| *Diag_anapat#Autre* | |  |  |  |  |  |

AFOP: Acute fibrinous and organising pneumonia; DAD: Diffuse alveolar damage

*Data were missing for eight patients.

**e-Figure 2: Distribution of the main histopathological diagnoses (n=155 patients)**


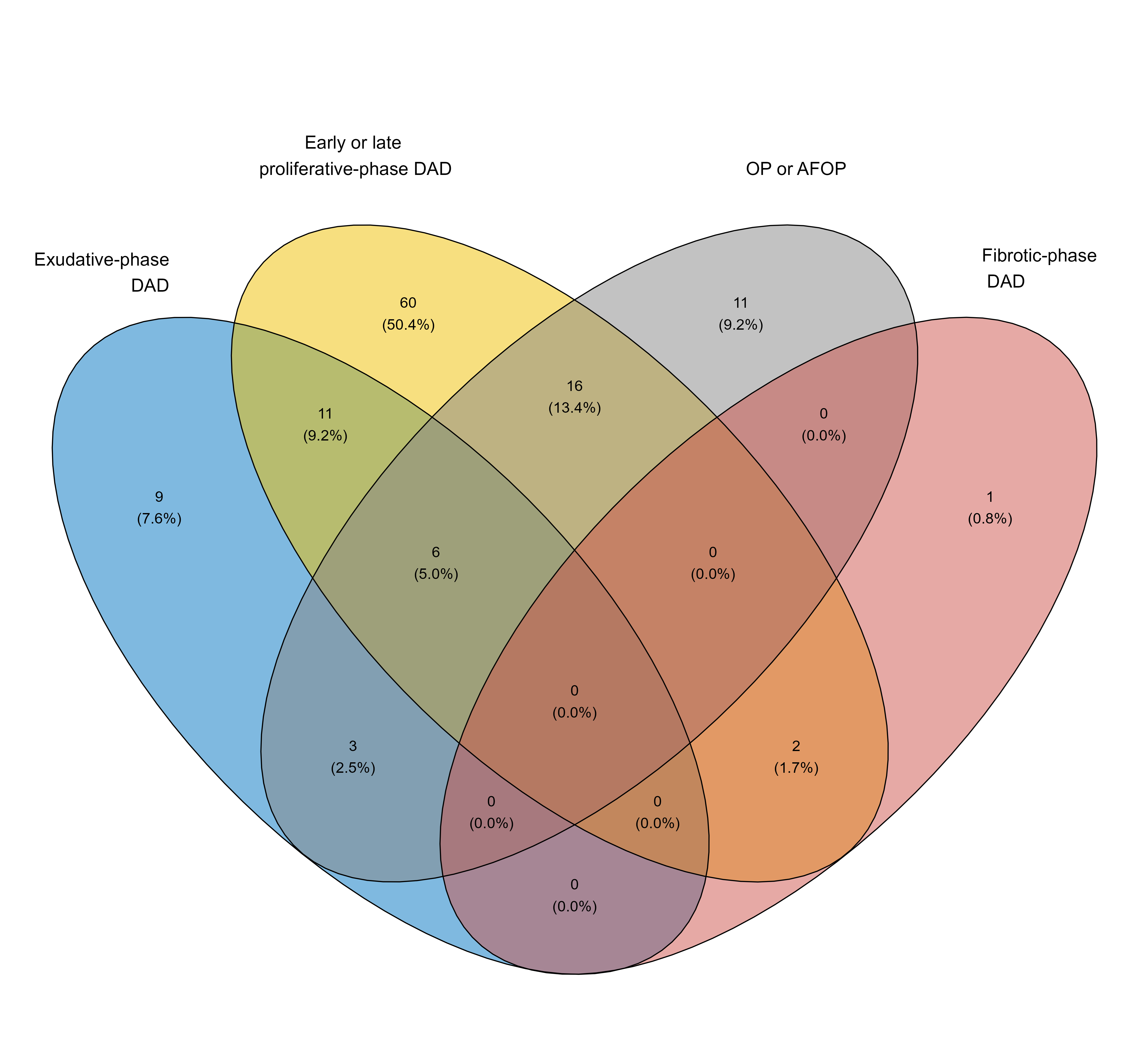


AFOP: acute fibrinous and organising pneumonia; DAD: diffuse alveolar damage; OP: organising pneumonia

**REFERENCES**

1. Brusselaers N, Lagergren J. The Charlson Comorbidity Index in Registry-based Research. Methods Inf Med. 2017;56(5):401–6.

2. ARDS Definition Task Force, Ranieri VM, Rubenfeld GD, Thompson BT, Ferguson ND, Caldwell E, et al. Acute respiratory distress syndrome: the Berlin Definition. JAMA. 2012 Jun 20;307(23):2526–33.

3. Section 2: AKI Definition. Kidney International Supplements. 2012 Mar;2(1):19–36.

4. European Centre for Disease Prevention and Control [Internet]. 2017 [cited 2022 May 26]. Surveillance of healthcare-associated infections and prevention indicators in European intensive care units: HAI-Net ICU protocol, version 2.2. Available from: https://www.ecdc.europa.eu/en/publications-data/surveillance-healthcare-associated-infections-and-prevention-indicators-european

5. Katzenstein AL, Bloor CM, Leibow AA. Diffuse alveolar damage--the role of oxygen, shock, and related factors. A review. Am J Pathol. 1976 Oct;85(1):209–28.

6. Tomashefski JF. Pulmonary pathology of acute respiratory distress syndrome. Clin Chest Med. 2000 Sep;21(3):435–66.

7. Beasley MB. The pathologist’s approach to acute lung injury. Arch Pathol Lab Med. 2010 May;134(5):719–27.

8. Beasley MB, Franks TJ, Galvin JR, Gochuico B, Travis WD. Acute fibrinous and organizing pneumonia: a histological pattern of lung injury and possible variant of diffuse alveolar damage. Arch Pathol Lab Med. 2002 Sep;126(9):1064–70.

9. The Lille COVID-19 ICU and Anatomopathology Group, Copin MC, Parmentier E, Duburcq T, Poissy J, Mathieu D. Time to consider histologic pattern of lung injury to treat critically ill patients with COVID-19 infection. Intensive Care Med [Internet]. 2020 Apr 23 [cited 2020 May 3]; Available from: http://link.springer.com/10.1007/s00134-020-06057-8

10. Koehler P, Bassetti M, Chakrabarti A, Chen SCA, Colombo AL, Hoenigl M, et al. Defining and managing COVID-19-associated pulmonary aspergillosis: the 2020 ECMM/ISHAM consensus criteria for research and clinical guidance. The Lancet Infectious Diseases. 2020 Dec;S1473309920308471.
